# Insignificant Medium-Term Vitamin D Status Change after 25-Hydroxyvitamin D Testing in a Large Managed Care Population

**DOI:** 10.1371/journal.pone.0105571

**Published:** 2014-08-19

**Authors:** Meng Wei, Run Yu, Stephen C. Deutsch

**Affiliations:** 1 Cedars-Sinai Medical Care Foundation, Beverly Hills, California, United States of America; 2 Division of Endocrinology, Cedars-Sinai Medical Center, Los Angeles, California, United States of America; University of Tennessee, United States of America

## Abstract

**Objectives:**

To examine the clinical utility of 25-hydroxyvitamin D (25(OH)D) testing in achieving medium-term vitamin D (VD) sufficiency in a managed care population.

**Methods:**

Retrospective study of a continuously-enrolled patient population in a 3-year period between 2011 and 2013. Primary outcome was VD status at ∼1 year after 25(OH)D testing. Patient demographics, comorbidities, medications, and 25(OH)D test results were gathered from relevant databases and multivariate logistic regression analysis used to study the risk factors of persistent VD deficiency or insufficiency.

**Results:**

Of 22,784 patients, 7533 (females 69.3%) did 14,563 25(OH)D tests, with an estimated cost of $582,520. Of the 7533 patients, 1126 had another 25(OH)D test at 300–400 days after the first one. Based on the two test results, 234 patients (20.8%) maintained sufficient 25(OH)D levels; 132 (11.7%) turned from VD-sufficient into VD-insufficient or -deficient; 538 (47.8%) remained VD-insufficient or -deficient, and only 222 (19.7%) improved to be VD-sufficient. Overall, only 8.0% more patients were VD-sufficient at ∼1 year after 25(OH)D testing. Only younger age and higher BMI were independent risk factors for persistent low 25(OH)D levels and high-dose VD use was not associated with achieving VD sufficiency.

**Conclusions:**

25(OH)D testing only benefits a small portion of patients thus lacks clinical utility in achieving VD sufficiency in the medium term but incurs a significant cost. A practical strategy to treat VD deficiency or insufficiency is needed; without it, 25(OH)D testing adds little value to most patients’ health and should be used with discretion.

## Introduction

Extremely low levels of vitamin D (VD), measured by serum 25-hydroxyvitamin D (25(OH)D) levels, are detrimental to bone health and VD supplementation in patients with very low 25(OH)D levels is beneficial in correcting osteomalacia or osteoporosis [1], [2]. There have been many studies suggesting that higher 25(OH)D levels are associated with better outcomes of numerous non-skeletal diseases such as cardiovascular diseases and cancers [3], [4]. Although there is no consensus on who should undergo 25(OH)D testing and routine screening for VD status is not recommended due to lack of evidence on feasibility, cost-effectiveness, or benefits of such screening [1], [2], some physicians may be convinced that it is worthwhile to screen for VD deficiency and to treat patients with VD if they do have it. In developed countries, 25(OH)D testing and the associated cost, mostly for screening purpose, have been dramatically increased over the last decade [5], [6]. To justify a screening test, the discovered condition has to be treatable if found earlier in its course. In the case of 25(OH)D testing, the discovered condition, VD deficiency (or insufficiency), is theoretically very treatable by sun exposure and VD supplements. Our clinical experience suggests that treatment of VD deficiency or insufficiency, however, is challenging in current clinical practice, especially if the VD status is followed in longer term [7]. The few prospective randomized clinical trials on VD supplementation, on the other hand, have not demonstrated consistent benefits in fracture risk reduction in community-dwelling individuals [1], [2], [8]–[11].

To test our hypothesis that most patients with VD deficiency (or insufficiency) remain VD-deficient (or -insufficient), we did a retrospective review of 25(OH)D testing and its clinical utility in achieving medium-term VD sufficiency in a managed care population in the 3 years between 2011 and 2013. The results of our study demonstrate that 25(OH)D testing lacks clinical utility in achieving VD sufficiency and should not be routinely performed in managed care populations or in other contemporary clinical practice.

## Methods

### Patients and 25(OH)D tests

This study has been approved by the Cedars-Sinai Institutional Review Board. Requirement of written or verbal consent was waived by the Review Board for this retrospective study. Health records were de-identified prior to use. All managed care patients who were continuously enrolled at a large medical group and an independent physician association from January 1, 2011 to December 31, 2013 were eligible. There were no exclusion criteria for this population-based study. The list of patients was obtained from the managed care enrollment database, race and body mass index (BMI) from the electronic medical records database, and 25(OH)D testing data from the laboratory encounter database. VD status was defined as follows: deficiency (25(OH)D≤20 ng/ml), insufficiency (>20 but <30 ng/nl), and sufficiency (≥30 ng/ml) [2]. The economic burden of 25(OH)D testing was calculated as the total cost of 25(OH)D testing within the study patient population in the 3 years of study period, based on the Medicare rate.

### Co-morbidities, procedures, and medications

Data on osteoporosis, pathologic fractures, obesity, fat malabsorption (including Crohn’s disease, ulcerative colitis, celiac disease, cystic fibrosis, and history of pancreatectomy, gastrectomy, or small bowel resection), chronic kidney disease, HIV, dual-energy X-ray absorptiometry (DEXA), and bariatric surgery were based on ICD-9 and CPT codes and obtained from encounter database. Medication records and high-dose VD dispensing (50,000 international units or 1.25 mg) data were obtained from pharmacy claim database. To study the risk factors for repeated 25(OH)D testing and failure to improve VD status after testing, the co-morbidities, procedures, and medications for osteoporosis and seizure recorded 6 months before and after the only or the first 25(OH)D test were used as variables. To study the risk factors for failure to improve VD status after testing, high-dose VD dispensed within 350 days after the only or the first 25(OH)D test was used.

### Statistics

Student t test was used to compare of the means of continuous data between two groups. Chi square or Fisher’s exact test was used to compare the rates of categorical data between groups. Multivariate logistic regression analysis was used to discover independent risk factors associated with a change of VD status [3], [12]. All statistical analyses were performed using the JMP Statistical Discovery software (Cary, NC).

## Results

**Figure 1:**
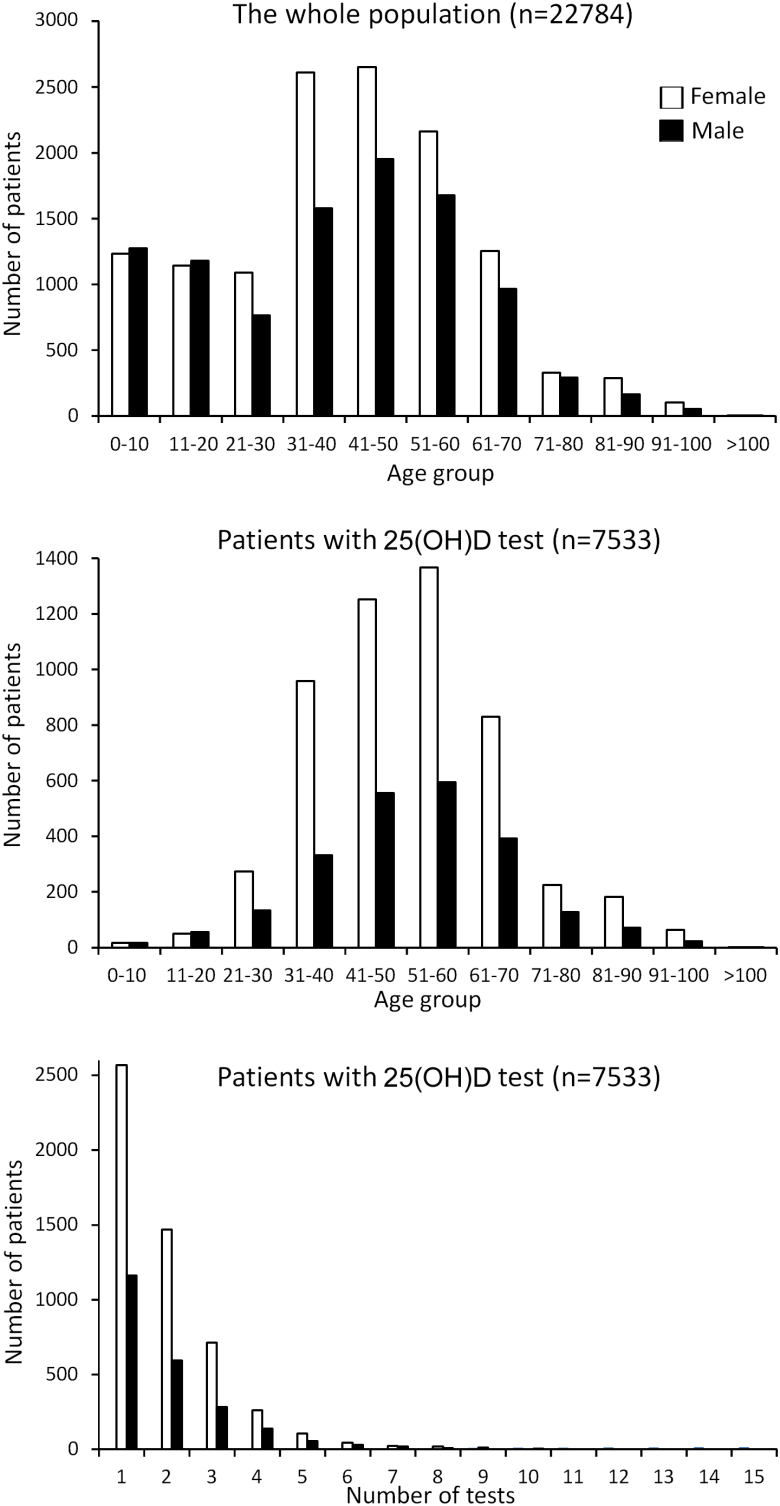
Demographics of the managed care population (Top) and the patients who underwent 25(OH)D testing (Middle) over a 3-year period. The numbers of patients who did multiple tests were also shown (Bottom).

Of the 22,784 managed care patients continuously enrolled in the 3-year study period (Fig. 1), 7533 patients did 14,563 25(OH)D tests with valid results, corresponding to an annual test rate of 11.0% of the managed care population and 0.21 25(OH)D test per patient per year. Based on Medicare rates, the total cost of the 25(OH)D tests for the 7533 patients was $582,520. Most tested patients were between 31 and 70 years old (83.4%), and those between 41 and 60 years old made up 50.0%. Females (n = 5224, 69.3%) were tested much more frequently than males (n = 2309, 30.7%) across all age groups. Of the 7533 patients, 3731 (49.5%) had only one 25(OH)D test but 3802 (50.5%) had two or more tests (Fig. 1). Compared with those with only 1 25(OH)D test, patients with more than one 25(OH)D test were 4.6 years older and had slightly higher 25(OH)D levels and BMI; they also had more osteoporosis diagnoses, underwent more DEXA scans, and were more likely on osteoporosis medications (Table 1). Overall, <3% of patients had history of bariatric surgery, fat malabsorption, chronic kidney disease, and HIV, or anti-epileptic use. More patients with more than one 25(OH)D test were dispensed with high-dose VD after the first 25(OH)D test than those with only one test (8.2% V 3.2%, *p*<0.001). Between the two groups, there were no significant differences in patients’ sex, race, rates of pathologic fractures, or obesity diagnosis.

**Table 1:** Comparison of patients who did only 1 25(OH)D test and those who did 2 or more 25(OH)D tests in the study period.

	Only 1 25(OH)D test	> = 2 25(OH)D tests
n	3,731	3,802
Age (range), yr	48.9 (3–105)***	53.5 (8–100)
Female, %	68.8	69.9
African American, %	22.1	21.5
Results of the only or first test(range), ng/ml	23.8 (0–115)***	26.0 (0–154.9)
Sufficiency/insufficiency/deficiency atthe only or first test, %	33.4/29.3/37.3***	31.8/34.3/33.9
Osteoporosis diagnosis, %	5.2***	9.3
Pathologic fractures, %	0.5	0.4
Osteoporosis medications, %	5.2***	8.9
DEXA, %	5.8***	11.1
Obesity diagnosis, %	14.3	15.8
BMI (range)	27.1 (13.4–66.6)***	27.6 (13.4–62.3)
BMI≥30, %	24.8	26.5
History of bariatric surgery, %	1.1**	1.9
Fat malabsorption, %	1.4	1.5
Chronic kidney disease, %	2.0*	2.8
HIV, %	0.9***	2.4
Anti-epileptics, %	0.3	0.2
High-dose VD prescription, %	3.2***	8.2

Statistical analysis was performed to assess the significance of the differences between the two groups.

*p<0.05,

**p<0.01,

***p<0.001.

**Figure 2:**
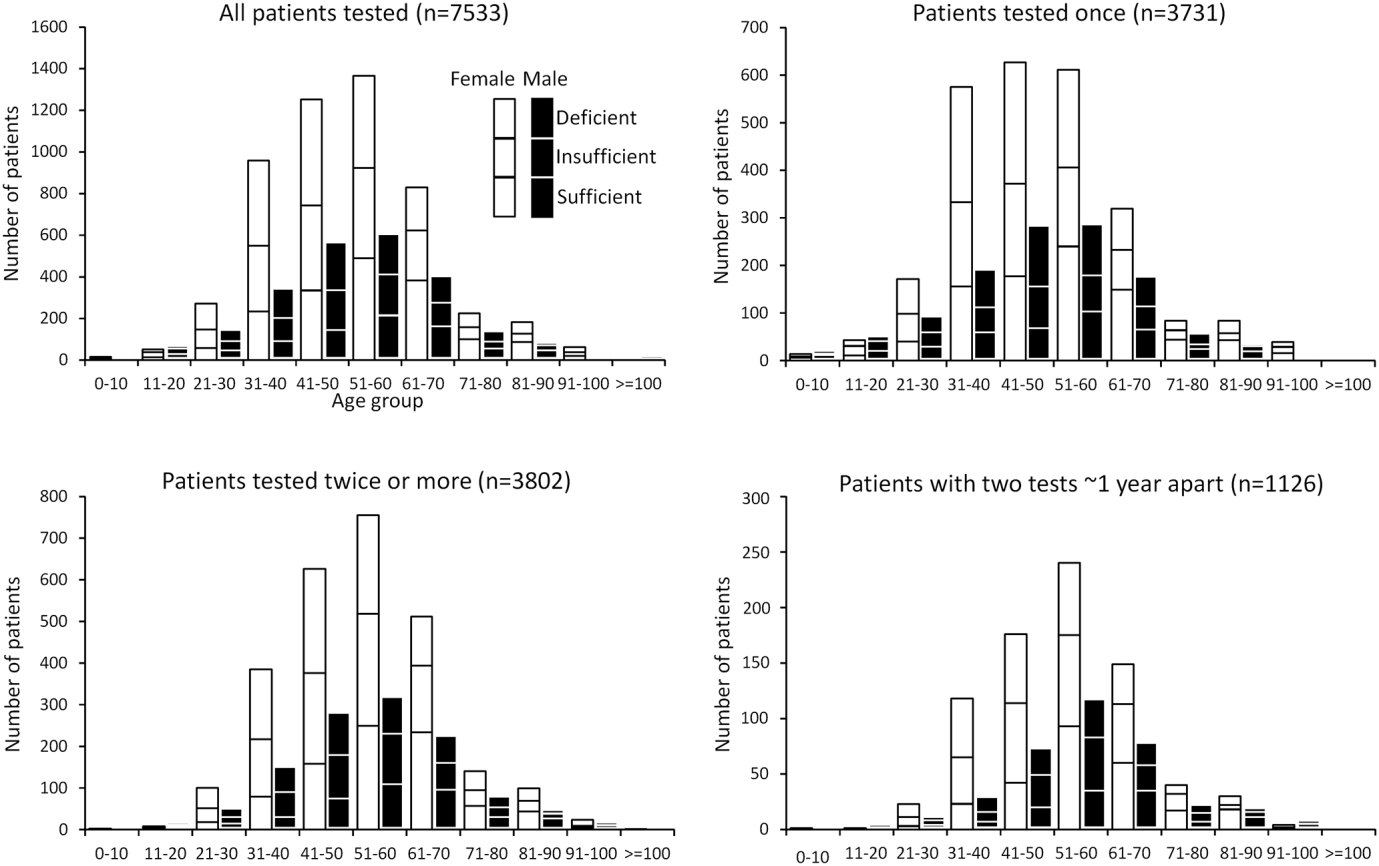
25(OH)D test results. The results of the only or the first 25(OH)D test were classified into 3 groups: deficiency (≤20 ng/ml), insufficiency (>20 but <30 ng/ml), or sufficiency (≥30 ng/ml). Upper left, results from all patients who underwent 25(OH)D testing. Upper right, results from the patients who had only been tested once. Lower left, results from the patients who had 2 or more 25(OH)D tests. Lower right, results from the patients who had another test at 300–400 days after the first one.

Based on the results of the first (or the only) 25(OH)D test, 32.6% of the 7533 patients were VD-sufficient while 31.6% VD-insufficient and 35.7% VD-deficient. Similar results were seen in both sexes across age groups (Fig. 2). Interestingly patients between 21 and 50 years old were less VD-sufficient (25.0% v. 39.2%, *p*<0.0001) but also less frequently tested than people of other ages (45.0% v. 55.2%, *p*<0.0001). There were small seasonal variations in 25(OH)D levels which were highest in July and lowest in December (mean levels 27.7 and 22.7 ng/ml, respectively). In the 21–50-year group, those who were VD-insufficient or -deficient were slightly more likely to be tested again than those who were VD-sufficient (46.3% v. 41.1%, *p* = 0.0077). In other age groups, the repeat test rates were not significantly different between those who were VD-insufficient or -deficient and those who were VD-sufficient (56.2% v. 53.7%, *p* = 0.1271).

To study the impact of 25(OH)D testing on future VD status, we identified 1126 patients who had done another 25(OH)D test at 300–400 days after the first one. The distribution of the results of the first 25(OH)D test of the 1126 patients and that of the 3802 patients were grossly similar to that of the 7533 patients (Fig. 2), suggesting that the 1126 patients were representative of all the patients tested more than once. We also compared the demographics and VD status of the 1126 patients with the rest of the 7533 (6507 patients, the difference of 7533 and 1026) over each decade between 21 and 90 years old. If the 25(OH)D results were divided into the 3 categories of sufficiency, insufficiency, and deficiency, the result distribution was not significantly different in most decades except for in the decades of 41–50 and 71–80 years old (*p* = 0.0196 and 0.0468, respectively). If the 25(OH)D results were divided into 2 groups, sufficiency and abnormally low levels (including insufficiency and deficiency), the 25(OH)D result distribution was not significantly different among any decades (*p* = 0.1272–0.9563).

**Figure 3:**
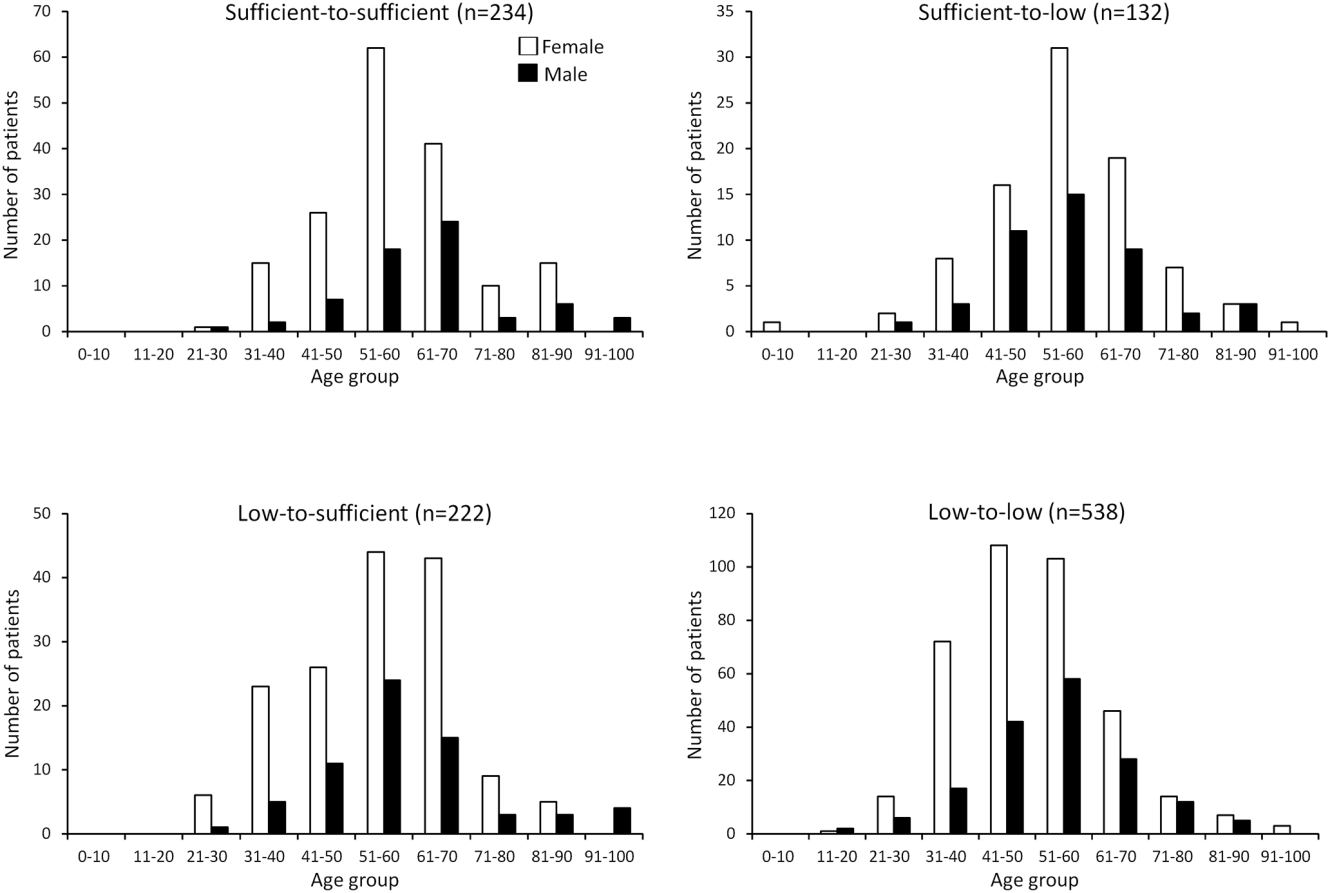
Demographics of the patients who had another 25(OH)D test at about 1 year after the first one. Upper left, the patients with sufficient 25(OH)D results from both tests (sufficient-to-sufficient). Upper right, the patients with sufficient 25(OH)D results from the first test but abnormally low results (VD-insufficient or deficient) from the second (sufficient-to-low). Lower left, the patients with abnormally low results from the first test but sufficient results from the second one (low-to-sufficient). Lower right, the patients with abnormally low results from both tests (low-to-low).

We compared the second and the first 25(OH)D test results of the 1126 patients and identified 4 patterns of VD status change. As the difference between VD deficiency and insufficiency is controversial and somewhat arbitrary [13], they were collectively labeled as abnormally low VD levels. Only 234 of the 1126 patients (20.8%) exhibited sufficient VD levels on both tests (sufficient-to-sufficient), indicating maintained VD sufficiency, while 132 (11.7%) who were originally VD-sufficient became VD insufficient or deficient (sufficient-to-low) (Table 2). Interestingly, after the first test, 538 patients (47.8%) remained VD-insufficient or -deficient (low-to-low) and only 222 patients (19.7%) improved to be VD-sufficient (low-to-sufficient). Overall, 67.5% of patients were VD-insufficient or -deficient at the first test and 59.5% were so at the second test. Thus only 8.0% patients benefited from the testing by converting from low to sufficient VD levels at approximately a year after the test. To derive risk factors for inability to maintain VD sufficiency and for persistent low VD levels, we compared the characteristics of the 4 groups of patients (Table 2 and Fig. 3). By univariate analysis, compared with those in the sufficient-to-sufficient group, the patients in the sufficient-to-low group were a few years younger, more obese, and with lower 25(OH)D levels from the first test; they also had fewer osteoporosis diagnoses and underwent fewer DEXA scans. Ironically, a small fraction of both groups of patients were dispensed with high-dose VD but those in the sufficient-to-low group were dispensed with relatively more frequently (6.1% v. 1.7%). Multivariate logistic regression analysis showed that only younger age and lower rate of HIV infection were independent risk factors for inability to maintain VD sufficiency. If only the significant variables identified by univariate analysis (*p*<0.05) were used, younger age remained the sole risk factor for inability to maintain VD sufficiency. Compared with those in the low-to-sufficient group, the patients in the low-to-low group were again a few years younger, more obese, and with lower 25(OH)D levels from the first test; more patients were African Americans. More patients in these two groups were dispensed with high-dose VD than those in the sufficient-to-sufficient and sufficient-to-low groups; however, patients in the low-to-low group were dispensed with relatively more high-dose VD than those in the low-to-sufficient group. Multivariate analysis showed that only younger age, higher BMI, history of bariatric surgery, and lower rate of HIV infection were independent risk factors for persistent low VD levels. If only the significant variables identified by univariate analysis (*p*<0.05) were used, younger age and higher BMI remained risk factors for persistent low VD levels.

**Table 2:** Comparison of 4 groups of patients who did another 25(OH)D test at ∼1 year after the first one.

Characteristics	Sufficient-to-sufficient	Sufficient-to-low	Low-to-sufficient	Low-to-low	All
n (%)	234 (20.8)	132 (11.7)	222 (19.7)	538 (47.8)	1126 (100)
Age (range), yr	59.2 (30–98)*	55.5 (8–91)	55.4 (21–95)***	51.4 (12–98)	54.3 (8–98)
Female, %	72.6	66.7	70.3	68.4	69.4
African American, %	16.9	18.1	12.6***	26.4	22.1
1^st^ result (range), ng/ml	43.1(30.0–154.9)***	37.0(30–87.2)	20.9(0–29.8)***	19.4(0–29.9)	26.7(0–154.9)
Difference between 2^nd^ and1^st^ results (range), ng/ml	−0.57(−110.2–38.1)***	−23.4(−69.8–−0.9)	17.6(0.5–81)***	−2.7(−29.7–28.0)	−0.7(−110.2–81)
Osteoporosis diagnosis, %	17.1*	9.1	5.4	5.8	9.2
Pathologic fractures, %	0.9	0	0	0.2	0.3
Osteoporosis medications,%	17.1	9.8	5.9	6.1	9.5
DEXA, %	20.9*	10.6	6.8	9.3	12.3
Obesity diagnosis, %	10.7	15.9	7.2***	19.1	16.1
BMI (range)	25.1(14.9–43.5)**	26.8(14.9–49.0)	26.7(16.9–47.6)***	28.7(16.5–62.3)	27.3(14.9–62.3)
BMI≥30, %	10.2**	21.6	15.2***	32.3	24.6
History of bariatric surgery, %	2.6	3.0	0.9	1.7	2.4
Fat malabsorption, %	3.0	3.0	0.5	1.1	1.6
Chronic kidney disease, %	3.4	3.8	2.3	3.3	3.5
HIV, %	6.4	2.3	2.3	1.1	2.9
Anti-epileptics, %	0	0.8	0	0.2	0.2
High-dose VD prescription, %	1.7*	6.1	5.0**	11.9	8.3

Sufficient-to-sufficient, patients with sufficient VD results from both tests; sufficient-to-low, patients with sufficient results from the first test but abnormally low results (VD-insufficient or deficient) from the second; low-to-sufficient, patients with abnormally low results from the first test but sufficient results from the second one; low-to-low, patients with abnormally low results from both tests. Statistical analysis was performed to assess the significance of the differences between the sufficient-to-sufficient and sufficient-to-low groups, and between the low-to-sufficient and low-to-low groups.

*p<0.05,

**p<0.01,

***p<0.001.

## Discussion

25(OH)D testing to detect VD deficiency has become a fad in recent years, probably driven by numerous correlational studies connecting low 25(OH)D levels and many human diseases [3]–[6]. As the literature on benefits of VD sufficiency appears somewhat plausible [1]–[4], one may presumptively accept that VD sufficiency is beneficial to health in general. The key question then becomes how to effectively help people achieve VD sufficiency. There is no doubt that high-dose VD treatment improves VD status in a few months [2], [3], [7] (and in years if given every 2 weeks [14]). As VD deficiency or insufficiency is a chronic condition, the key question is whether VD status improves in longer terms after 25(OH)D testing or high-dose VD treatment. Thus in this article, we take a novel approach studying the practical clinical utility of 25(OH)D testing and examine whether 25(OH)D testing helps achieve VD sufficiency in the medium term in a large managed care population.

First of all, the study confirms that VD sufficiency is uncommon (only ∼1/3) in the mostly adult managed care population [15]. The most striking finding, however, is that most patients with VD deficiency or insufficiency (∼70%) do not achieve VD sufficiency and a portion of patients with VD sufficiency (∼40%) lose it at 300–400 days after the first 25(OH)D test. Overall only 8% of the tested patients improve their VD status from deficiency or insufficiency to sufficiency. The true cost of 25(OH)D testing is thus more expensive than the apparent cost because to benefit one patient (to achieve or maintain VD sufficiency after 25(OH)D testing), 12.5 patients (the inverse of 8%) have to be tested. As 1/3 of the entire study population underwent 25(OH)D testing at least once and only <10% of tested patients had osteoporosis diagnosis or history of pathologic fractures, the purpose of the 25(OH)D tests was most likely for screening for VD deficiency. Our results seriously challenge the clinical utility of 25(OH)D testing for screening purpose. First, there does not seem to be a need for screening for VD deficiency or insufficiency if the majority of the population has the condition. Second, the lack of practical and effective treatment of VD deficiency or insufficiency makes VD status screening not worthwhile. Although chronic use of high-dose VD can achieve VD sufficiency for years, this method has not been adopted in clinical practice [14]. Indeed, routine screening for vitamin D status is not recommended by guidelines but the reason for not recommending screening is due to lack of evidence on feasibility, cost-effectiveness, or benefits of such screening [1], [2]. Our results provide direct evidence for recommending *against* routine screening for vitamin D status.

Although VD deficiency or insufficiency can be treated in a theoretically straightforward manner by sun exposure and VD supplement, our study clearly shows that it is challenging to be treated in practice. The failure to effectively treat VD deficiency or insufficiency may be either due to lack of counseling on importance and ways to achieve VD sufficiency or due to patients’ incompliance. Concern of skin cancer is a known barrier to achieve VD sufficiency [16], [17]. Adherence to VD supplementation is an important factor in achieving benefits of VD supplementation on fracture prevention [7], [11]. Adherence is satisfactory (60–90%) in elderly populations but unclear in younger populations. If VD sufficiency is indeed critical to health, a practical and effective method is needed to achieve VD sufficiency.

Our study provides novel insights into the potential causes of VD deficiency and failure to improve VD status. In our study, the patients who maintain VD sufficiency are not necessarily healthier than others. They are older and thinner, and tend to have osteoporosis and HIV infection. As most of them do not take high-dose VD supplement, their VD sufficiency is presumably achieved by sun exposure and over-the-counter VD supplement [2], [3]. As these measures are dependent on life style, it can be assumed that these patients are highly motivated to achieve VD sufficiency. Although we don’t have data on their motivations, their older age and the age-associated osteoporosis in this mostly female population may motivate them to achieve and maintain VD sufficiency. On the other hand, the patients who remain VD-deficient or -insufficient are younger and heavier but fewer of them have osteoporosis or HIV infection. Even though slightly more than 10% of them fill high-dose VD prescriptions, the high-dose VD does not help them achieve VD sufficiency in the medium term. Thus the cause of their persistent VD deficiency or insufficiency is also unconducive life style for VD sufficiency. Their perceived low risk for osteoporosis may give them a disincentive to make life style modifications to achieve VD sufficiency. Compared with other chronic life-style diseases such as obesity, diabetes, and hypertension, VD deficiency can be much more easily treated by simply taking daily 2000–4000 international units of VD without sun exposure [1], [2]. The lack of clear evidence that mild VD deficiency or insufficiency indeed causes clear and severe complications is likely the main reason for physicians and patients not to invest in life style modifications to improve VD status. On the other hand, for motivated patients, the physicians should consider the patients’ risk factors for persistent low VD levels during counseling; for example, higher doses of VD are likely needed for young and obese patients to achieve VD sufficiency [18]–[20].

Our study has its strengths and limitations. Because it is based on a population without exclusion criteria, its results more realistically reflect the true characteristics of the population. The defined and stable managed care patient population has several distinct advantages for population studies [21]. As the patients received all their care within the managed care system and their 25(OH)D tests and results, comorbidities, procedures, and medications were all recorded, our study results are accurate and unlikely prone to misinterpretation due to incomplete data. Most importantly, we have followed a cohort of the same patients with 2 25(OH)D tests approximately a year apart so that we can derive specific risk factors for failure to achieve or maintain VD sufficiency. One major limitation is that we do not have data on sun exposure and over-the-counter VD supplement, the main treatments for VD deficiency or insufficiency. Another limitation is that although the cohort of patients we followed appeared to be similar to all patients with 25(OH)D testing, the former has much fewer patients than, and may not represent a random sample of, the latter. The small seasonal variation of 25(OH)D levels should not affect our analysis on medium-term VD status because the second 25(OH)D test was carried out 1 year later, in the same season in which the first test was done.

In summary, this retrospective study of a continuously enrolled managed care population in a 3-year period demonstrates that 25(OH)D testing only benefits a small portion of patients thus lacks clinical utility in achieving VD sufficiency in the medium term but incurs a significant cost. Our data implies that a practical and effective strategy to treat VD deficiency or insufficiency is needed in contemporary managed care practice; without such as strategy, 25(OH)D testing adds little value to most patients’ health and should be used with discretion.

## References

[1] IOM (Institute of Medicine) (2011) Dietary reference intakes for calcium and vitamin D. Washington, DC: The National Academies Press; 2011. 21796828

[2] Holick MF, Binkley NC, Bischoff-Ferrari HA, Gordon CM, Hanley DA, et al (2011) Evaluation, treatment, and prevention of vitamin D deficiency: an Endocrine Society clinical practice guideline. J Clin Endocrinol Metab 96: 1911–1930. 21646368 10.1210/jc.2011-0385

[3] Holick MF (2007) Vitamin D deficiency. N Engl J Med 357: 266–281. 17634462 10.1056/NEJMra070553

[4] Basit S (2013) Vitamin D in health and disease: a literature review. Br J Biomed Sci 70: 161–72. 24400428 10.1080/09674845.2013.11669951

[5] Sattar N1, Welsh P, Panarelli M, Forouhi NG (2012) Increasing requests for vitamin D measurement: costly, confusing, and without credibility. Lancet 379: 95–96. 22243814 10.1016/S0140-6736(11)61816-3

[6] Bilinski KL, Boyages SC (2012) The rising cost of vitamin D testing in Australia: time to establish guidelines for testing. Med J Aust 197: 90. 10.5694/mja12.10561 22794049

[7] Yu R (2014) Commentary on “Surge in US Outpatient Vitamin D Deficiency Diagnoses: National Ambulatory Medical Care Survey Analysis”. South Med J 107: 218–219. 24937513 10.1097/SMJ.0000000000000094

[8] Jackson RD1, LaCroix AZ, Gass M, Wallace RB, Robbins J, et al (2006) Calcium plus vitamin D supplementation and the risk of fractures. N Engl J Med 354: 669–683. 16481635 10.1056/NEJMoa055218

[9] Sanders KM1, Stuart AL, Williamson EJ, Simpson JA, Kotowicz MA, et al (2010) Annual high-dose oral vitamin D and falls and fractures in older women: a randomized controlled trial. JAMA 303: 1815–1822. 20460620 10.1001/jama.2010.594

[10] Bischoff-Ferrari HA1, Willett WC, Orav EJ, Lips P, Meunier PJ, et al (2012) A pooled analysis of vitamin D dose requirements for fracture prevention. N Engl J Med 367: 40–49. 22762317 10.1056/NEJMoa1109617

[11] Reid IR, Bolland MJ, Grey A (2014) Effects of vitamin D supplements on bone mineral density: a systematic review and meta-analysis. Lancet 383: 146–155. 24119980 10.1016/S0140-6736(13)61647-5

[12] Looker AC, Pfeiffer CM, Lacher DA, Schleicher RL, Picciano MF, et al (2008) Serum 25-hydroxyvitamin D status of the US population: 1988–1994 compared with 2000–2004. Am J Clin Nutr 88: 1519–1527. 19064511 10.3945/ajcn.2008.26182 PMC2745830

[13] Rosen CJ (2011) Clinical practice. Vitamin D insufficiency. N Engl J Med 364: 248–254. 21247315 10.1056/NEJMcp1009570

[14] Pietras SM, Obayan BK, Cai MH, Holick MF (2009) Vitamin D2 treatment for vitamin D deficiency and insufficiency for up to 6 years. Arch Intern Med 26: 1806–8. 10.1001/archinternmed.2009.361 19858440

[15] Forrest KY, Stuhldreher WL (2011) Prevalence and correlates of vitamin D deficiency in US adults. Nutr Res 31: 48–54. 21310306 10.1016/j.nutres.2010.12.001

[16] Reeder AI1, Jopson JA, Gray AR (2012) “Prescribing sunshine”: a national, cross-sectional survey of 1,089 New Zealand general practitioners regarding their sun exposure and vitamin D perceptions, and advice provided to patients. BMC Fam Pract 13: 85. 22901028 10.1186/1471-2296-13-85 PMC3460728

[17] Bonevski B, Girgis A, Magin P, Horton G, Brozek I, et al (2012) Prescribing sunshine: a cross-sectional survey of 500 Australian general practitioners' practices and attitudes about vitamin D. Int J Cancer. 130: 2138–2145. 10.1002/ijc.26225 21647876

[18] Lee P, Greenfield JR, Seibel MJ, Eisman JA (2009) Center JR (2009) Adequacy of vitamin D replacement in severe deficiency is dependent on body mass index. Am J Med 122: 1056–1060. 19854337 10.1016/j.amjmed.2009.06.008

[19] Stratton-Loeffler MJ, Lo JC, Hui RL, Coates A, Minkoff JR, et al (2012) Treatment of vitamin D deficiency within a large integrated health care delivery system. J Manag Care Pharm 18: 497–505. 22971203 10.18553/jmcp.2012.18.7.497 PMC10438277

[20] Drincic A, Fuller E, Heaney RP, Armas LA (2013) 25-hydroxyvitamin D response to graded vitamin D3 supplementation among obese adults. J Clin Endocrinol Metab 98: 4845–4851. 24037880 10.1210/jc.2012-4103

[21] Vogt TM1, Elston-Lafata J, Tolsma D, Greene SM (2004) The role of research in integrated healthcare systems: the HMO Research Network. Am J Manag Care 10: 643–648. 15515997

